# Metabolic and microbial signatures in rat hepatocellular carcinoma treated with caffeic acid and chlorogenic acid

**DOI:** 10.1038/s41598-017-04888-y

**Published:** 2017-07-03

**Authors:** Zhan Zhang, Di Wang, Shanlei Qiao, Xinyue Wu, Shuyuan Cao, Li Wang, Xiaojian Su, Lei Li

**Affiliations:** 1 Department of Hygiene Analysis and Detection, School of Public Health, Nanjing Medical University, 101 Longmian Avenue, Nanjing, 211166 Jiangsu P. R. China; 2 Nanjing entry-exit inspection and quarantine bureau, 110 Jiangjun Avenue, Nanjing, 211106 Jiangsu P.R. China

## Abstract

Hepatocellular carcinoma (HCC) treatment remains lack of effective chemopreventive agents, therefore it is very attractive and urgent to discover novel anti-HCC drugs. In the present study, the effects of chlorogenic acid (ChA) and caffeic acid (CaA) on HCC induced by diethylnitrosamine (DEN) were evaluated. ChA or CaA could reduce the histopathological changes and liver injury markers, such as alanine transarninase, aspartate aminotransferase, alkaline phosphatase, total bile acid, total cholesterol, high density lipoprotein cholesterol and low density lipoprotein cholesterol. The underlying mechanisms were investigated by a data integration strategy based on correlation analyses of metabonomics data and 16 S rRNA gene sequencing data. ChA or CaA could inhibit the increase of *Rumincoccaceae UCG-004* and reduction of *Lachnospiraceae incertae sedis*, and *Prevotella 9* in HCC rats. The principal component analysis and partial least squares discriminant analysis were applied to reveal the metabolic differences among these groups. 28 different metabolites showed a trend to return to normal in both CaA and ChA treatment. Among them, Bilirubin, L-Tyrosine, L-Methionine and Ethanolamine were correlated increased *Rumincoccaceae UCG-004* and decreased of *Lachnospiraceae incertae sedis* and *Prevotella 9*. These correlations could be identified as metabolic and microbial signatures of HCC onset and potential therapeutic targets.

## Introduction

Hepatocellular carcinoma (HCC) is a global problem and the second most common cause of cancer related deaths in the world^1, 2^. Primary prevention of HCC can be achieved with universal vaccination against Hepatitis B virus (HBV) infection^3^. Owing to their characteristics of high chemical diversity and biochemical specificity, a broad spectrum of phytochemicals including flavonoids, alkaloids and polyphenols, has been isolated and investigated for anti-HBV activities^4^. Chlorogenic acid (ChA) and caffeic acid (CaA) are abundant polyphenol compounds in the human diet^5, 6^. CaA is a hydrlysed metabolite of ChA by mucosal and/or microbial esterase in the intestinal tract^7^. Our previous study had revealed they could protect against polychlorinated biphenyls or tetrachloride-induced hepatotoxicity^8^. In addition, tea polyphenols provided an effective and promising alternative for the chemoprevention and treatment of HCC^9^. Tea polyphenols epigallocatechin gallete and the aflavin could restrict mouse liver carcinogenesis through modulation of self-renewal Wnt and hedgehog pathways^10^. However, the role of ChA or CaA in prevention of HCC is still unclear. And elucidating the underlying mechanisms is crucial for the development of effective strategies to prevent HCC.

The gut and liver are key organs in nutrient absorption and metabolism. The unique immunological environment in the liver has been attributed to its close connection to the gut. The portal vein delivers gut-derived products, such as lipopolysaccharide (LPS), bacterial DNA and peptidoglycan, to the liver. The commensal microbiota plays a beneficial role in maintaining liver homeostasis and preventing liver fibrosis^11^. Imbalance of the gut microbiota is associated with hepatic diseases, such as lipid accumulation, stellate cell activation, immune cell recruitment^12^. Growing evidence indicated that gut microbiota was involved in initiation, progression and therapy of HCC^13, 14^. Our previous study had shown that CaA could restore the reduction of richness of fecal microbiota in experimental colitis^15^. The effects of CaA or ChA on composition of gut microbiota associated with HCC remain unclear.

Metabolomics is a rapidly evolving technology for identifying novel biomarkers by assessing large numbers of metabolites that are substrates and products in metabolic pathways. In addition, it is also a new avenue for understanding of cellular and organism specific responses to environment, chemicals and drugs perturbations^16^. The metabolic features of HCC included elevated glycolysis, gluconeogenesis, and β-oxidation with reduced tricarboxylic acid cycle^17^. Identification of metabolites may highlight how CaA or ChA affect HCC. As well known, gut microbiota is exclusively responsible for several metabolic important functions, such as short chain fatty acid production, bile acid biotansformation, hydrolysis and fermentation of non-digestible substrates^18^. Indeed, the metabolomics approach has been applied to several studies on the gut microbiota, mostly focused on the exploration of disease-related metabolites in order to obtain detailed information on the gut metabolic pathways^19^. In the present study, the effects of CaA and ChA on HCC induced by diethylnitrosamine (DEN) were evaluated. The functional relationships between metabolites and gut microbial composition was investigated by a data integration strategy based on correlation analyses of serum metabonomics data and 16 S rRNA gene sequencing data.

## Results

### Body weight and Histopathological observations

**Figure 1:**
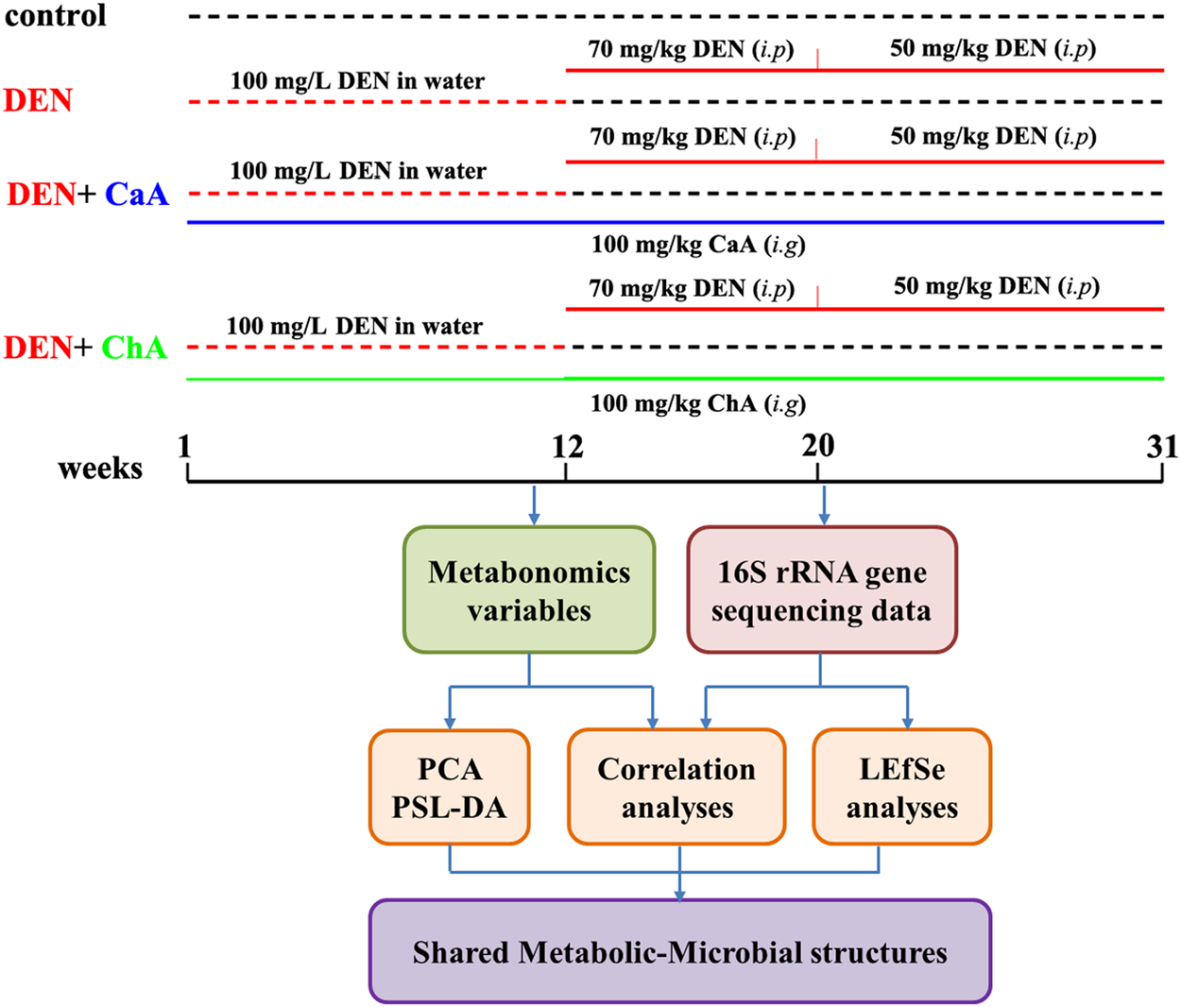
Overview of the experimental design and the analytical strategy.

**Figure 2:**
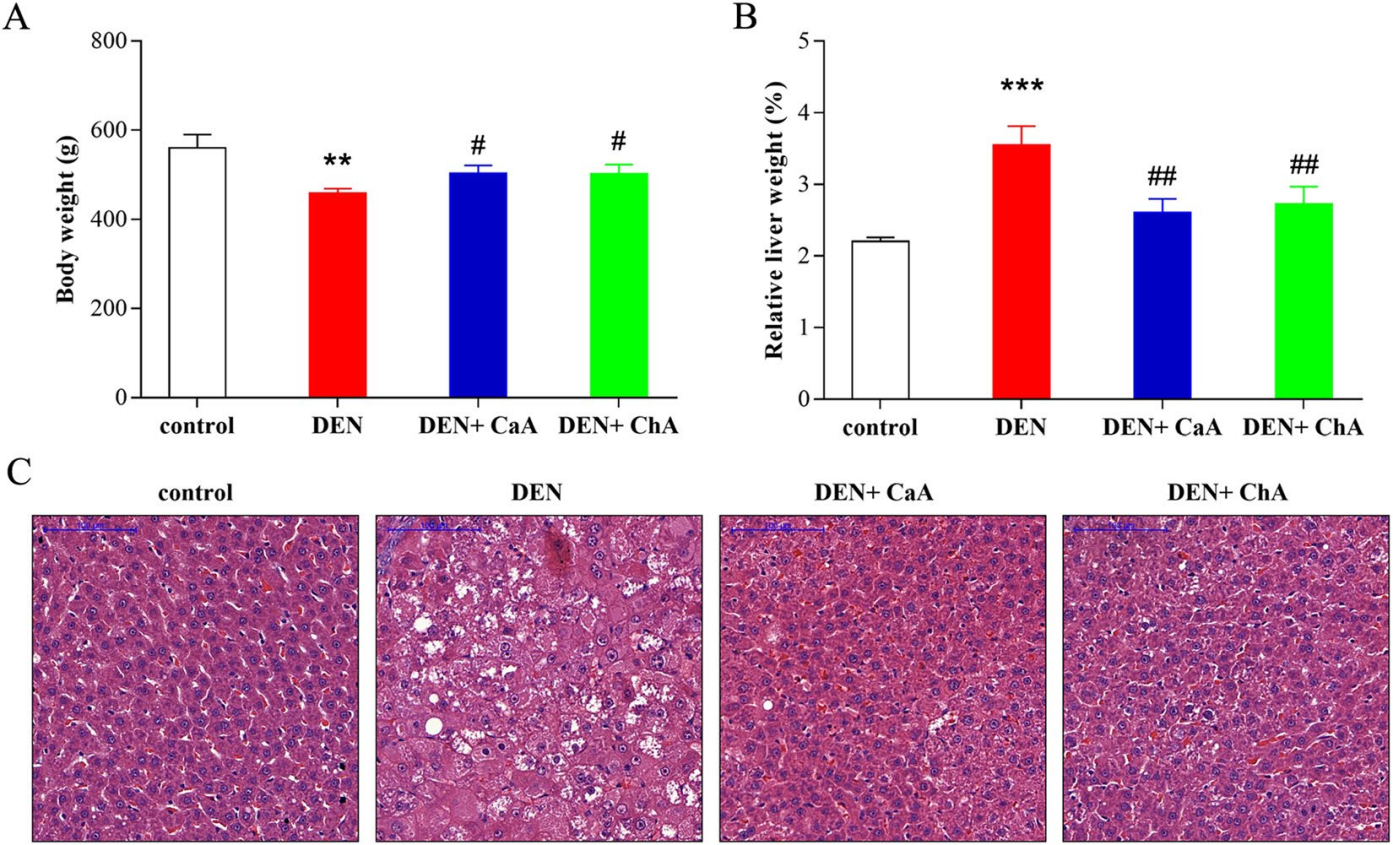
CaA and ChA improved DEN induced HCC in rats. (**A**) Body weight, relative and (**B**) relative liver weight were expressed as mean ± SD of 6 rats in each group. **P < 0.01, ***P < 0.001 compared with control group; ^#^ *P* < 0.05, ^##^ *P* < 0.01 compared with DEN group. (**C**) Representative images of the liver tissues from control, DEN, DEN + CaA and DEN + ChA group. Formalin fixed, paraffin-embedded 5 μm cross-sections were stained with H&E. Scale bar: 100 μm.

A schematic diagram of the experimental study design was shown in Fig. 1. Compared with the control group, the body weight of DEN group significantly decreased (Fig. 2A). Moreover, DEN treatment significantly increased the relative liver weight. Co-treatment with DEN and CaA or ChA could inhibit the decrease of body weight and the increase of the relative liver weight (Fig. 2B). The liver section from control group showed normal architecture and hepatic cells with granulated cytoplasm, small uniform nuclei and nucleolus (Fig. 2C). HCC group showed loss of architecture and neoplastic cells arranged in lobules. Neoplastic cells were larger than normal cells with granular cytoplasm and larger hyperchromatic nuclei and hyaline globules. CaA or ChA treated group showed nearly normal architecture only with few malignant hepatocytes.

### Biochemical analyses

**Figure 3:**
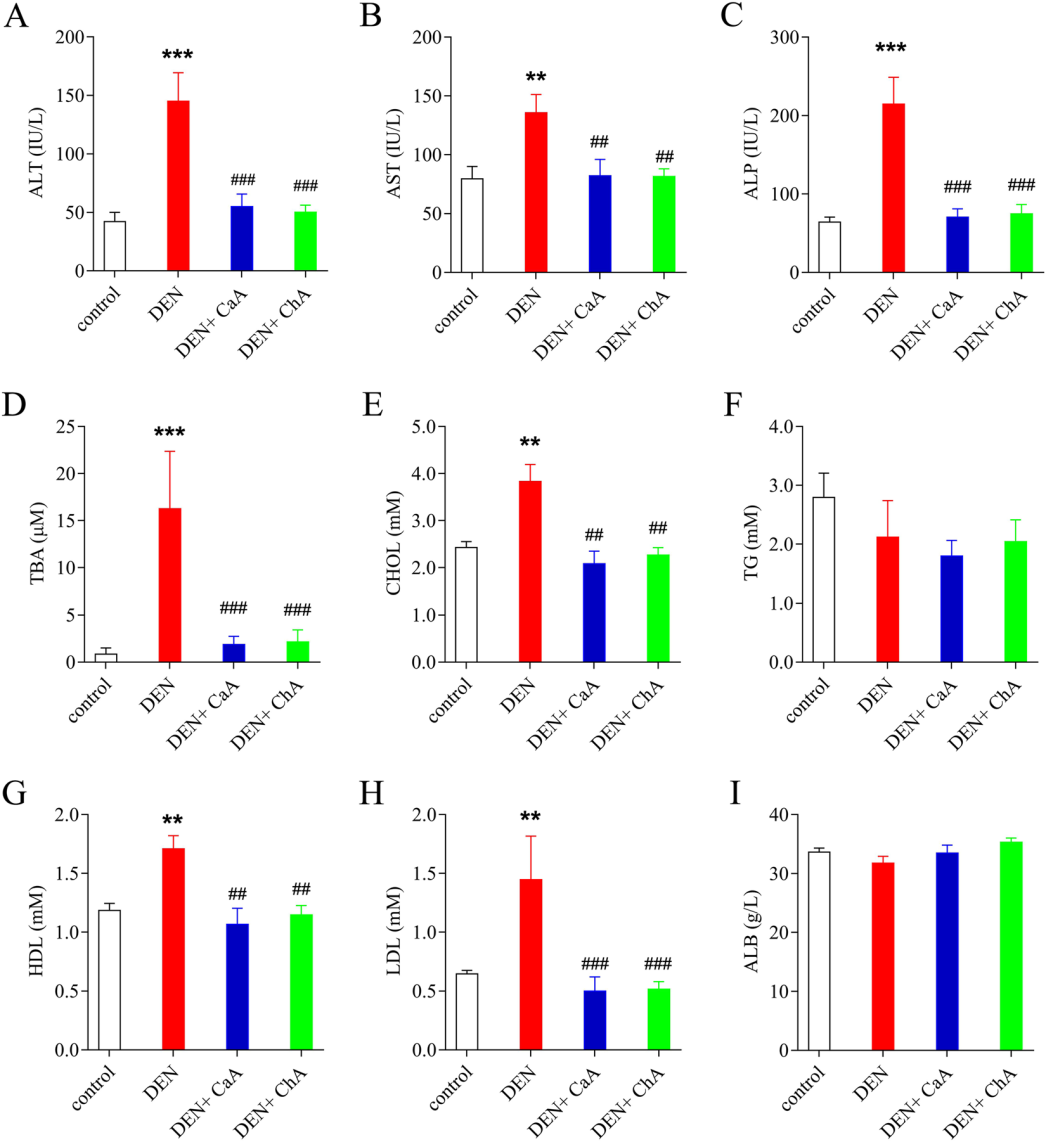
The effects of CaA and ChA on serum biochemical parameters in HCC induced by DEN. (**A**) alanine transarninase (ALT), (**B**) aspartate aminotransferase (AST), (**C**) alkaline phosphatase (ALP), (**D**) total bile acid (TBA), (**E**) total cholesterol (TC), (**F**) triglycerides (TG), (**G**) high density lipoprotein cholesterol (HDL), (**H**) low density lipoprotein cholesterol (LDL) and (**I**) albumin (ALB) were assessed. Data were expressed as mean ± SD of 6 rats in each group. **P < 0.01, ***P < 0.001 compared with control group; ^##^ *P* < 0.01, ^###^ *P* < 0.001 compared with DEN group.

The serum biochemical parameters basically supported the results obtained from histopathological examinations. There was a significant elevation in serum levels of ALT (Fig. 3A), AST (Fig. 3B), ALP (Fig. 3C), TBA (Fig. 3D), CHOL (Fig. 3E), HDL (Fig. 3G) and LDL (Fig. 3H) and in the DEN group in contrast to the control group. Co-treatment with DEN and CaA or ChA significantly decreased serum levels of ALT, AST, ALP, TBA, CHOL, HDL and LDL. However, no significant changes of the LPS levels in serum or liver were observed in DEN groups (Fig. S1).

### Effects of CaA or ChA on fecal microbiota in DEN-treated rats

**Figure 4:**
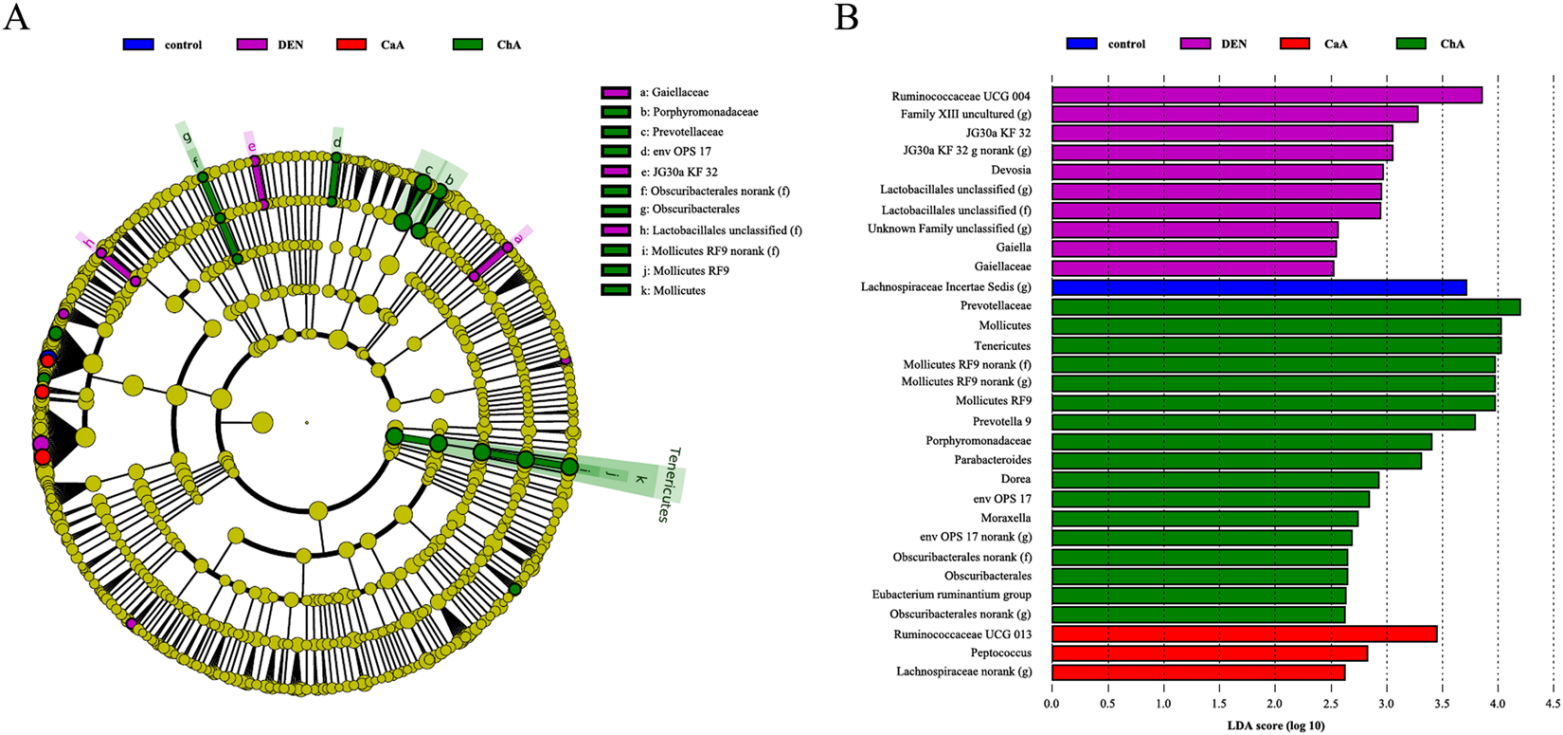
Discriminative taxa of fecal microbiota among control, DEN, DEN + CaA and DEN + ChA group. (**A**) Cladogram representing the features that are discriminative among these groups using the LDA model results on the bacterial hierarchy. Significantly discriminant taxon nodes are colored and branch areas are shaded according to the highest-ranked variety for that taxon. For each taxon detected, the corresponding node in the taxonomic cladogram is colored according to the highest-ranked group for that taxon. If the taxon does not show significantly differential representation between sample groups, the corresponding node is colored yellow. (**B**) LDA coupled with effect size measurements identifies the most differentially abundant taxons among these groups.

**Figure 5:**
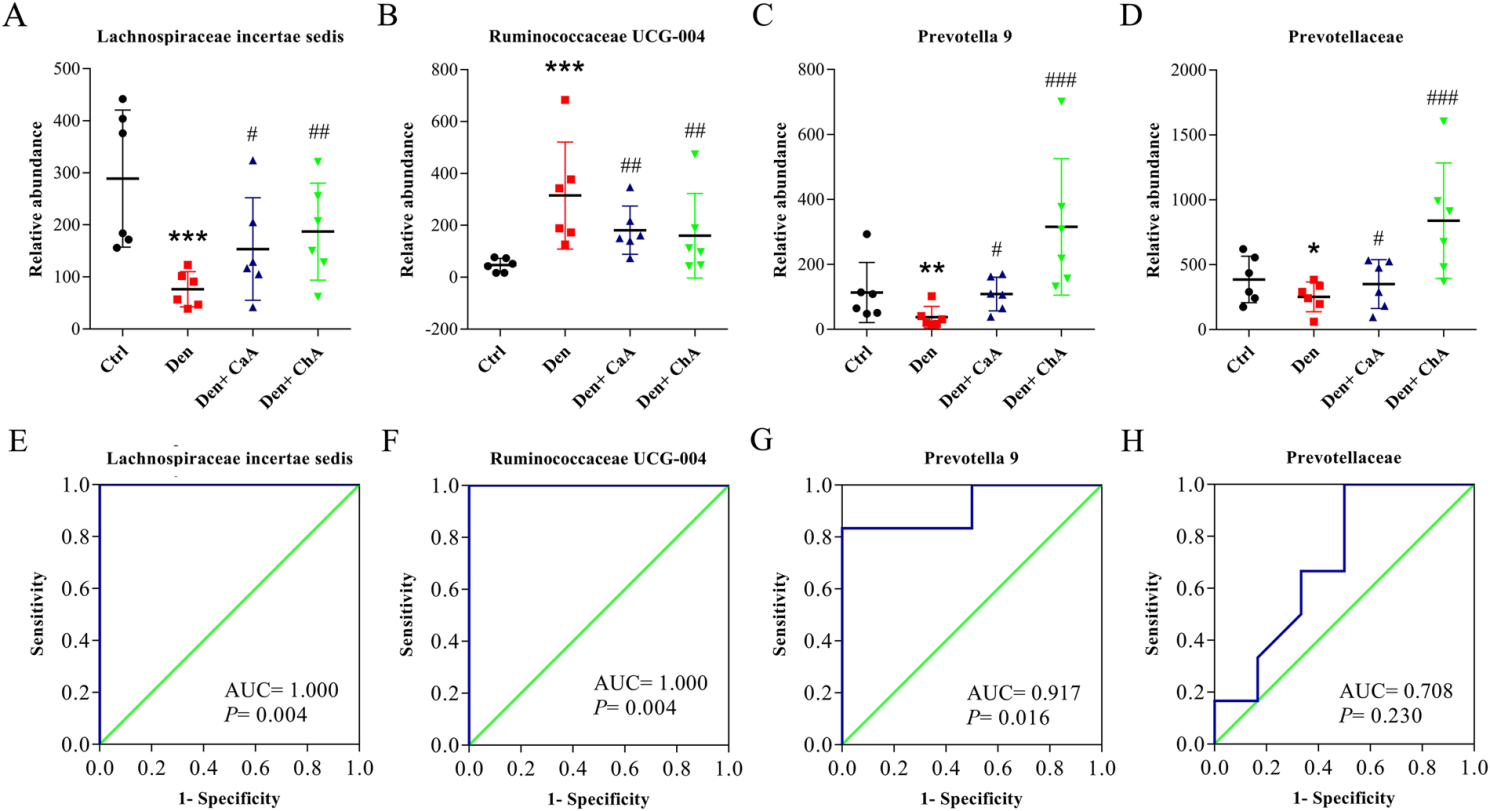
Taxonomic differences of fecal microbiota among control, DEN, DEN + CaA and DEN + ChA group. Comparsion of relative of (**A**) *Lachnospiraceae incertae sedis*, (**B**) *Rumincoccaceae UCG-004*, (**C**) *Prevotella 9* and (**D**) *Prevotellaceae* among these groups. The statistical difference was analyzed by Mann-Whitney U test. *P < 0.05, **P < 0.01, ***P < 0.001 compared with control group; ^#^ *P* < 0.05, ^##^ *P* < 0.01, ^###^ *P* < 0.001, compared with DEN group. Receiver operating characteristic (ROC) curves for (**E**) *Lachnospiraceae incertae sedis*, (**F**) *Rumincoccaceae UCG-004*, (**G**) *Prevotella 9* and (H) *Prevotellaceae* were used to predict HCC.

Compared with the control group, no significant changes of the richness or diversity of fecal microbiota were observed in DEN, CaA or ChA group (Fig. S1). To identify the specific bacterial taxa associated with HCC, we compared the fecal microbiota among these groups using LEfSe (Fig. 4A). The greatest differences in taxa among the four communities are displayed, and several genera could be used as distinguishing biomarkers (Fig. 4B). The changes in the fecal microbiota among these four groups were explored using the Mann-Whitney U test. *Lachnospiraceae incertae sedis* (Fig. 5A), *Prevotella 9* (Fig. 5C) and *Prevotellaceae* (Fig. 5D) were significantly less abundant in the fecal microbiota of DEN group compared the control group, while *Rumincoccaceae UCG-004* (Fig. 5B) was significantly more abundant in the fecal microbiota of DEN group. CaA or ChA treatment could significantly reverse these effects. The ROC curves indicated that *Lachnospiraceae incertae sedis* (Fig. 5E), *Rumincoccaceae UCG-004* (Fig. 5F) or *Prevotella 9* (Fig. 5G) could be used as a microbial signature for the differential diagnosis of HCC.

### Metabolic profiles and correlation analysis between the metabolite and microbiota

**Figure 6:**
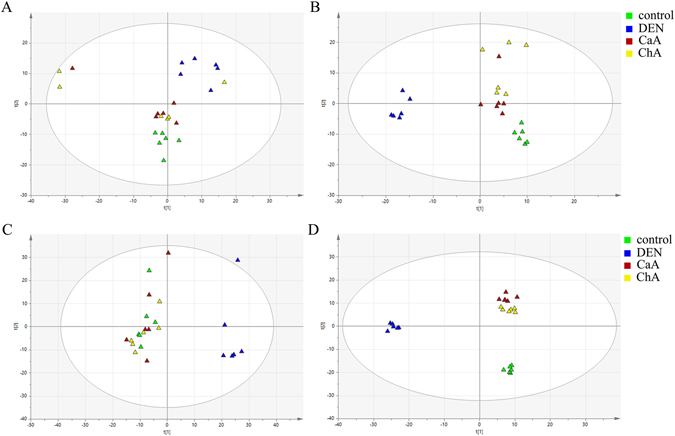
PCA and PSL-DA score scatter plots show metabolic pattern among control, DEN, DEN + CaA and DEN + ChA group. (**A**) PCA and (**B**) PLS-DA score plot conducted by GC-MS data (R2Y = 0.952, Q2 = 0.411). (**C**) PCA and (**D**) PLS-DA score plot conducted by HPLC-MS data (R2Y = 0.995, Q2 = 0.443).

**Figure 7:**
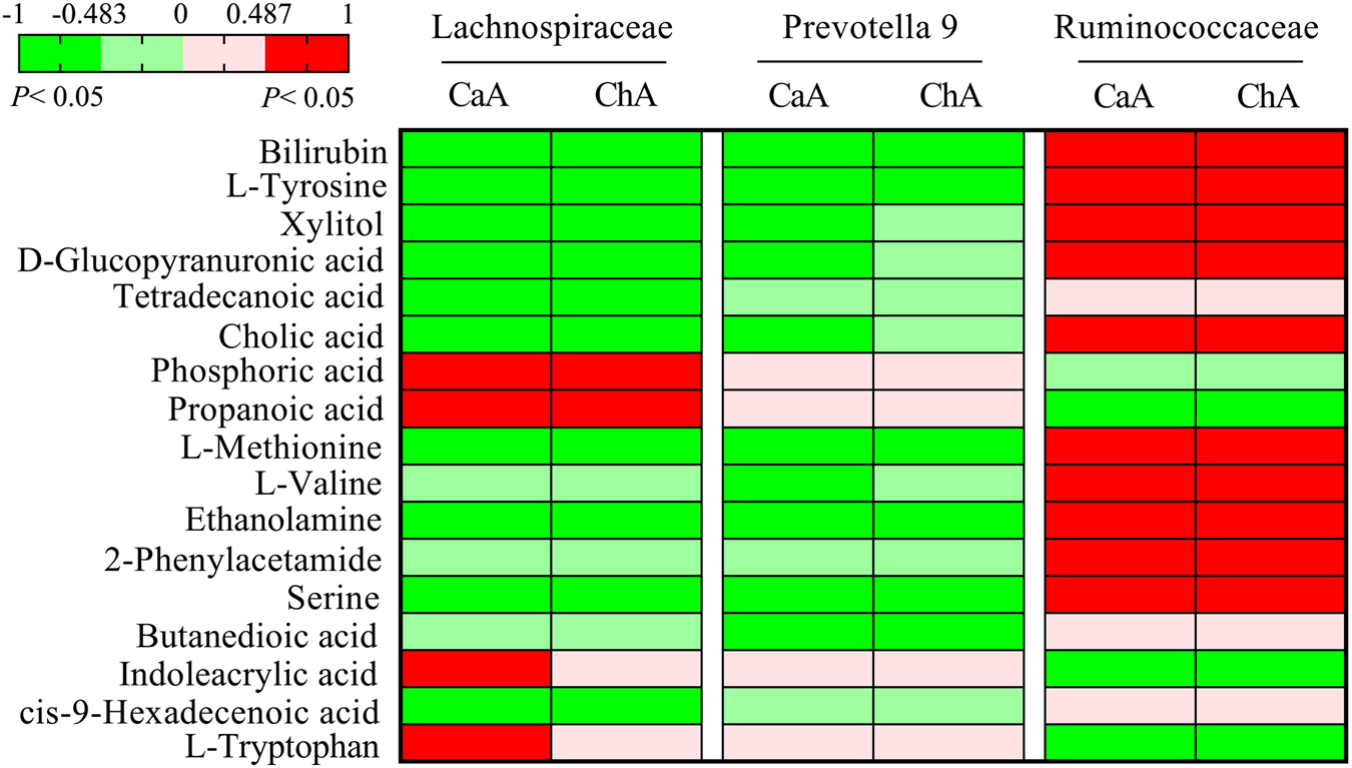
Heatmap with color gradients related to correlation coefficient between the variables. A red cell represents a significant correlation between the corresponding metabolite and microbiota component; green represents a significant anticorrelation between the variables; and other cells indicated no significant correlation. The correlation coefficients were assessed in CaA (control, DEN and DEN + CaA) and ChA (control, DEN and DEN + ChA) group, respectively.

The score plot showed a relative good separation between DEN group and other three groups which preliminarily indicated the different metabolic pattern between groups (Fig. 6A,C). The score plots of PLS-DA model also showed a clearly discrimination between DEN group and other three groups (Fig. 6B,D). The cluster of CaA or ChA group was apparently moving towards to that of control group which was consistent with histopathological and biochemical observations, suggesting they could improve HCC. The VIP and t test analyses identified 72 metabolites that were significantly different between controls and DEN groups (Table. S1). There were 41 and 37 significantly different metabolites turned out a trend to return to normal after CaA and ChA treatment, respectively. Among them, 28 metabolites were significantly different in both CaA and ChA groups compared with the DEN groups (Table 1). They could be the biomarkers to evaluate the effects of CaA or ChA from the perspective of metabolomics. Furthermore, the correlation between these 28 metabolites and *Prevotella 9*, *Lachnospiraceae incertae sedis* or *Rumincoccaceae UCG-004* were investigated. 17 metabolites were correlated with at least one of them in both CaA and ChA groups (Fig. 7). Bilirubin, L-Tyrosine, L-Methionine and Ethanolamine were correlated with all these three bacteria.

**Table 1:** List of candidate biomarkers for HCC and evaluating the effects of CaA and ChA.

Metabolites	m/z	VIP score	DEN vs control		CaA vs DEN		ChA vs DEN		Corresponding metabolic pathway
			fold	p	fold	p	fold	p	
Glycerophosphocholine	257.1028	2.28	1.35	0.024	1.47	0.003	1.29	0.015	Glycerophospholipid metabolism
Bilirubin	584.2635	2.18	8.32	<0.001	0.44	<0.001	0.39	<0.001	Porphyrin and chlorophyll metabolism
L-Tyrosine	181.0739	2.16	1.65	<0.001	0.62	<0.001	0.59	<0.001	Phenylalanine, tyrosine and tryptophan biosynthesis, Phenylalanine metabolism
Xylitol	307	2.01	8.26	<0.001	0.42	<0.001	0.46	<0.001	Pentose and glucuronate interconversions
Isopropyl-beta-D-thiogalactopyranoside	361	1.90	0.71	<0.001	1.33	<0.001	1.37	<0.001	Carbohydrate Metabolism
D-Glucopyranuronic acid	292	1.90	4.32	<0.001	0.44	<0.001	0.39	<0.001	
Tetradecanoic acid	285	1.87	1.86	<0.001	0.65	<0.001	0.71	0.005	energy metabolism
Cholic acid	408.2876	1.86	9.28	0.002	0.21	0.001	0.22	0.001	Primary bile acid biosynthesis
Phosphoric acid	299	1.84	0.36	<0.001	2.59	<0.001	2.24	<0.001	phospholipid metabolism
Propanoic acid	174	1.82	0.66	<0.001	1.19	0.003	1.23	0.002	intestinal flora metabolism
L-Methionine	176	1.81	1.22	<0.001	0.84	<0.001	0.85	<0.001	Aminoacyl-tRNA biosynthesis
L-Valine	117.079	1.80	1.51	0.001	0.69	0.001	0.68	0.001	Valine, leucine and isoleucine biosynthesis
Ethanolamine	174	1.80	1.34	<0.001	0.76	<0.001	0.79	0.001	Glycerophospholipid metabolism
L-Histidine	155.0695	1.76	1.17	0.021	0.88	0.035	0.79	0.001	Aminoacyl-tRNA biosynthesis
2-Phenylacetamide	135.0684	1.74	1.42	<0.001	0.75	0.001	0.72	<0.001	Phenylalanine metabolism
D-Mannose	387	1.55	0.38	0.003	2.07	0.002	2.31	0.005	Galactose metabolism
Campesterol	382	1.47	0.33	0.006	2.07	0.001	2.74	<0.001	Biosynthesis of terpenoids and steroids
Pentanoic acid	200	1.46	0.68	0.007	1.48	0.004	1.39	0.017	intestinal flora metabolism
Glutamic acid	246	1.42	1.26	0.009	0.72	0.001	0.84	0.015	Tyrosine metabolism
d-Fructose	315	1.41	0.42	0.015	1.85	0.011	2.08	0.036	Fructose and mannose metabolism
Serine	204	1.40	1.20	0.011	0.80	0.001	0.83	0.006	Glycine, serine and threonine metabolism, Methane metabolism
Pentanedioic acid	198	1.38	1.57	0.012	0.56	0.002	0.63	0.003	amino acid metabolism
Butanedioic acid	233	1.38	1.33	0.013	0.60	<0.001	0.69	<0.001	Citrate cycle (TCA cycle)
Indoleacrylic acid	187.0633	1.35	0.89	0.002	1.13	0.005	1.16	0.013	Tryptophan metabolism
cis-9-Hexadecenoic acid	311	1.31	1.44	0.019	0.63	0.001	0.72	0.010	fatty acid metabolism
meso-Erythritol	217	1.26	1.17	0.026	0.83	0.001	0.78	<0.001	
Maltose	361	1.24	0.44	0.029	1.40	0.002	1.47	0.032	Carbohydrate digestion and absorption
L-Tryptophan	204.0899	1.23	0.89	0.009	1.11	0.005	1.13	0.033	Tryptophan metabolism, Aminoacyl-tRNA biosynthesis

## Discussion

Hepatocellular carcinoma (HCC) is a common malignancy worldwide, with ~600,000 newly diagnosed cases, and leading to > 250,000 mortalities annually^1, 20^. DEN is a potent hepatocarcinogenic nitrosamine that could induce lesion as well as tumors in rodents with marked biochemical, histological and molecular similarity to the progression of HCC in humans^21, 22^. Although, no chemotherapy agent has better benefit than surgery due to different reasons, including drug resistance and toxicity to normal cells^23^. The development of innovative novel therapeutics for the management of HCC is particularly urgent. Accumulating evidence has demonstrated that CaA and ChA possessed various pharmacological effects, such as antioxidative, antiviral, antitumor, and anti-inflammatory activities^24–26^. The aim of this study was to evaluate the effects of CaA and ChA on HCC induced by DEN. To the best of our knowledge, this is the first study using an integrated metabonomics and 16 S rRNA gene sequencing data to investigate gut microbiota signatures of HCC onset and potential therapeutic targets for CaA and ChA.

Accumulative evidence indicated that intestinal microbiota played important role in the pathogenesis of HCC. Previous study had shown significant structural alterations in gut microbiota during the development of HCC, and *Atopobium spp*., *Bacteroides spp*., *Bacteroides vulgatus*, *Bacteroides acidifaciens*, *Bacteroides uniformis*, *Clostridium Desulfovibrio spp*., *cocleatum* and *Clostridium xylanolyticum* were increased^27^. And endotoxin level was increased in DEN induced HCC in rats^13^. However, no significant changes of diversity and richness of fecal microbiota or endotoxin were observed in the present study. LEfSe and ROC analysis revealed that *Lachnospiraceae incertae sedis*, *Rumincoccaceae UCG-004* and *Prevotella 9* could be used as a microbial signature for the differential diagnosis of HCC. Recent study indicated *Lachnospiraceae* could discriminate non-alcoholic fatty liver disease (NAFLD) patients from controls. And increased *Ruminococcus* could be a gut microbiota signature of NAFLD onset and NAFLD-NASH (non-alcoholic steatohepatitis) progression^28^. *Prevotella* is a beneficial bacterium that can produce short chain fatty acids. Prohep, a novel probiotic mixture, could suppress HCC growth in mice by regulation of the T cells differentiation in the gut, which in turn alters the level of the proinflammatory cytokines in the tumor microenvironment^29^. In the present study, CaA or ChA treatment could reverse the decrease of *Prevotella 9*.

As well known, most of the gut microorganisms are anaerobe, such as *Lachnospiraceae* and *Prevotella*. Our previous study had shown that CaA or ChA exert antioxidative activity^8^, their strong oxygen radical scavenging capacity may provide a survival advantage of these bacteria. In addition, CaA and ChA had been shown to possess antibacterial activity^30^, which could be associated with a reduction in the abundance of species capable of holding these bacteria, thus favouring a rise in their proportion. However, we have not directly established the causal relationship between the changes of gut microbiota and dietary CaA or ChA.

HCC encompasses a spectrum of hepatic pathology. Similar to previous study^31^, levels of serum ALT and AST were increased in DEN group. CaA or ChA treatment remarkably inhibit the activities of these enzymes, and these effects could be confirmed by histopathological observation. TBA was increased in cirrhotic and HCC patients, and bile acids could also promote DEN-induced HCC via increased inflammatory signaling^32^. HCC patients with low level of ALP have favorable overall survival^33^. In the present study, CaA or ChA treatment could significantly block the increase of TBA and ALP in DEN group. The disorder of lipid metabolism is the major hallmark of HCC^34^. CHOL, HDL and LDL were increased in DEN group, CaA or ChA treatment could significantly reverse these effects. CaA and ChA may have indirect effect of on liver function through gut microbiota.

Characteristic metabolic deregulations of HCC may enable novel biomarkers discovery for early diagnosis. In the present study, 72 differential metabolites with statistical significance were firstly acquired based on the comparison between DEN group and the control. To pick up the same therapeutic targets of CaA or ChA, these differential metabolites were further explored based on the comparison between DEN group and CaA or ChA group. Further, the metabolite-microbial relationships were evaluated by the correlation analyses. The initial data was composed of 28 metabolites and 3 microbiota variables. 17 metabolites were correlated with at least one of 3 microbiota in both CaA and ChA groups. Bilirubin, L-Tyrosine, L-Methionine and Ethanolamine were increased in DEN group, and they were correlated with all these three bacterium. Dietary L-Methionine restriction has been reported to improve hepatocyte function in mammals^35^. The aberrant ethanolamine phospholipid metabolism in cancer has recently further been established and solidified as a universal metabolic hallmark of cancer^36^. In HCC patients, elevated bilirubin levels were associated with higher AFP levels, increased portal vein thrombosis and multifocality and lower survival^37^. Oxidized tyrosine enhanced AST and ALT activities, increased total bilirubin content, and led to oxidative damage in rat liver^38^. In addition to liver, gut microbiota could influence hepatic metabolites and plasma biomarkers, such as HDL cholesterol, free fatty acids, and bilirubin^39^. The metabolic process of L-Tyrosine is carried out by bacteria as well by liver cells^40^. Tyrosine could be metabolized by *Lachnospiraceae* and *Rumincoccaceae in vitro* ^41^. *Prevotella processes* methionine γ lyase, which could catalyze α, γ-elimination of L-methionine^42^.

The integration of metabonomics and fecal microbiota profiling provided important information on the changes of gut microbiota and metabolites in DEN induced HCC after CaA or ChA treatment. The correlation between gut microbiota and metabolites provided important information on the development of HCC, which could also be potential therapeutic targets. Further human studies are required to validate the proposed model and to better describe other associations between gut microbiota phylotypes, metabotypes, and disease phenotypes.

## Methods

### Chemicals and materials

HPLC grade methanol, acetonitrile and formic acid were all purchased from Merck (Merck, Darmstadt, Germany). Diethylnitrosamine (DEN, ≥99.0% purity) was purchased from CNW Tecnologies (Anpel, Shanghai, China). Caffeic acid (purity >99%) was purchased from Aladdin (Shanghai, China). Chlorogenic acid (purity >99%) was purchased from Nanjing ZhiBaiCui Biology Technology Co., Ltd (Nanjing, China). N,O-Bis(trimethylsilyl)trifluoroacetamide (BSTFA) was obtained from Sigma-Aldrich (St. Louis, MO, USA). Deionized water was manufactured by Milli-Q50SP Reagent system (Millipore Corporation, MA, USA). The enzyme linked immunosorbent assay (ELISA) kits of rat LPS was obtained from SenBeiJia Biotechnology Co., Ltd. (Nanjing, China).

### Animal-experiments and sample preparation

Twenty four male Wistar rats (150~200 g) were supplied by Shanghai SLAC Lab. Animal Co., Ltd. (Shanghai, China). The rats were housed in a breeding room with temperature of 22–24 °C, relative humidity of 50–70%, and a 12 h light-dark cycle. They were fed with standard pellet diet, supplied with water ad libitum, and were acclimatized to the facilities 1 week prior to the experiments. The rat HCC model was established by DEN treatment according to previous study with some modified^43^. These rats were divided into 4 groups: control group, DEN group, CaA group and ChA group. The control group received tap water, CaA group and ChA group received 100 mg/kg CaA and ChA by intragastric (*i.g*.) administration throughout the experiment, respectively. Meanwhile, DEN group, CA group and ChA group rats were given water containing 100 mg/L DEN from week 1 to week 12. These three groups were administrated with DEN (70 mg/kg) via intraperitoneal injection (*i.p*.) twice a week from week 13 to week 20. These three groups were given 50 mg/kg DEN (*i.p*.) every three days from week 21 to week 31. After sacrificed, blood, tissue and stool samples were collected and stored in −80 °C before use. The care and use of the animals were followed the animal welfare guidelines, and all the experimental protocols were approved by the Animal Care and Welfare Committee of Nanjing Medical University.

### Determination of biochemical parameters

Serum biochemical parameters, such as alanine transarninase (ALT), aspartate aminotransferase (AST), albumin (ALB), alkaline phosphatase (ALP), total bile acid (TBA), total cholesterol (TC), triglycerides (TG), high density lipoprotein cholesterol (HDL) and low density lipoprotein cholesterol (LDL) were measured by a biochemistry analyzer (Hitachi 7100, Japan). The levels of LPS in serum and liver were determined using ELISA kits.

### Histological analysis

The anterior portion of the left lateral lobe of the liver was sectioned and used for histological analysis. The tissue was fixed by immersion in 10% neutral-buffered formalin. The sample was then embedded in paraffin, sliced into 5 ìm sections and stained with hematoxylin and eosin (H&E), followed by blinded histological assessment using an optical microscope (Axioskop 2 plus, Carl Zeiss, Hamburg, Germany).

### Gut microbiota characterization

Genomic DNA was extracted from each fecal sample according to our previous study^15^. Bacterial 16 S rRNA at the V3 hypervariable region was amplified using a set of primers (338 F: 5′-GTGCCAGCMGCCGCGGTAA-3′ and 806 R: 5′-GGACTACHVGGGTWTCTAAT-3′). Sequencing was performed by an Illumina MiSeq (PE300). Sequences were then trimmed and classified with the QIIME toolkit. The high-quality reads were clustered into operational taxonomic units (OTUs) using Mothur. The OTUs that reached at a 97% nucleotide similarity level were used for alpha diversity (Shannon and Simpson index), richness (ACE and Chao1), Good’s coverage, and rarefaction curve analysis using Mothur. Taxonomy-based analyses were performed by classifying each sequence using the Naïve Bayesian Classifier program of the Michigan State University Center for Microbial Ecology Ribosomal Database Project (RDP) database (http://rdp.cme.msu.edu/) with a 70% bootstrap score. LEfSe (http://huttenhower.sph.harvard.edu/galaxy/) was used to identify taxa that differed consistently between sample types according to previous studies^44, 45^.

### Metabolic profiling

Thawed serum smaples (450 μl) were spiked with ice cold methanol (1350 μl). After centrifugation of the mixture at 12000 rpm g for 15 min at 4 °C, the supernatant fraction was collected and divided into two parts: one (300ìl) for liquid chromatography- mass spectrometry (LC-MS) analysis and one (150 ìl) for gas chromatography -mass spectrometer (GC-MS) analysis after derivatization with BSTFA (containing 1% TMCS). Serum metabolic profiling analysis by LC-MS was performed as our previous study^46^. Briefly, LC-MS analysis was performed on a Thermo Scientific Q Exactive hybrid quadrupole-orbitrap mass spectrometer coupled with a UPLC Ultimate 3000 system (Thermo Fisher Scientific, Bremen, Germany) equipped a heated electrospray source (HESI), at both positive and negative ion modes. LC-MS analysis was performed on a Thermo TRACE 1310 gas chromatograph system coupled with a Thermo TSQ 8000 Triple Quadrupole mass spectrometer. The quality control (QC) was pooled with same volume from each sample to ensure the reproducibility and stability druing the whole procedure.

### Metabolomics Data processing and Statistical analysis

The GC-MS data files were processed by R software package and the UPLC-MS data files were performed via SIEVE software (Thermo Fisher Scientific) for peak feature extraction, migration time correction, denoising, smoothing, alignment and normalization. A total of 3172 features were extracted from the LC-MS data (2891 from positive ion mode and 281 from negative ion mode), and 831 were extracted from the GC-MS data. After data pretreatment, the processed data were subjected to multivariate statistical analysis using SIMCA-P 13.0 software (Umetrics, Umea, Sweden)^47^. The principal component analysis (PCA) and the partial least squares discriminant analysis (PLS-DA) were performed to detect distributions of deferent groups, classification and comparison in each group. The variable importance in the projection (VIP) obtained from multivariate statistical analysis can provided significantly changed variables after drug intervention. The statistical significance was calculated using the Student t-test (*P* value < 0.05), in summary, the variates which *P* value less than 0.5 with VIP value larger than 1.0 were considered as significative differential metabolites^48^. The identification of different signal from MS data was using an in-house metabolite library, literatures and database, like HMDB. The pathway analysis was performed by MetaboAnalyst 3.0 (http://www.metaboanalyst.ca)^49^ and KEGG database was also used to identify relevant metabolic pathway.

### Statistical analysis

The differences in body weight, relative liver weight and biochemical parameters were analyzed using one-way analysis of variance (ANOVA) by SPSS 13.0 software (Chicago, IL, USA). The receiver operating characteristic (ROC) curve for each crucial taxa was generated, and the area under the parametric curve (AUC) was computed by SPSS. A Mann-Whitney U test was used to assess the differences in taxonomy of fecal microbiota. The correlation between richness of fecal microbiota and significant differential metabolites were analyzed by R software. Differences were considered statistically significant at *P* ≤ 0.05.

## Electronic supplementary material

Supplementary information

Table S1
