# Supplementary material for: Metabolic and microbial signatures in rat hepatocellular carcinoma treated with caffeic acid and chlorogenic acid
Source: Sci Rep. 2017 Jul 3;7:4508. doi: 10.1038/s41598-017-04888-y (PMC5495756; doi:10.1038/s41598-017-04888-y)
Supplement: Supplementary file 1 — Supplementary information [file 41598_2017_4888_MOESM1_ESM.doc]

**Metabolic and microbial signatures in rat hepatocellular carcinoma treated with caffeic acid and chlorogenic acid**

Zhan Zhang^1a^, Di Wang^1a^, Shanlei Qiao^1^, Xinyue Wu^1^, Shuyuan Cao^1^, Li Wang^1^, Xiaojian Su^2^, Lei Li^1🖂^

**Supplementary information**

**
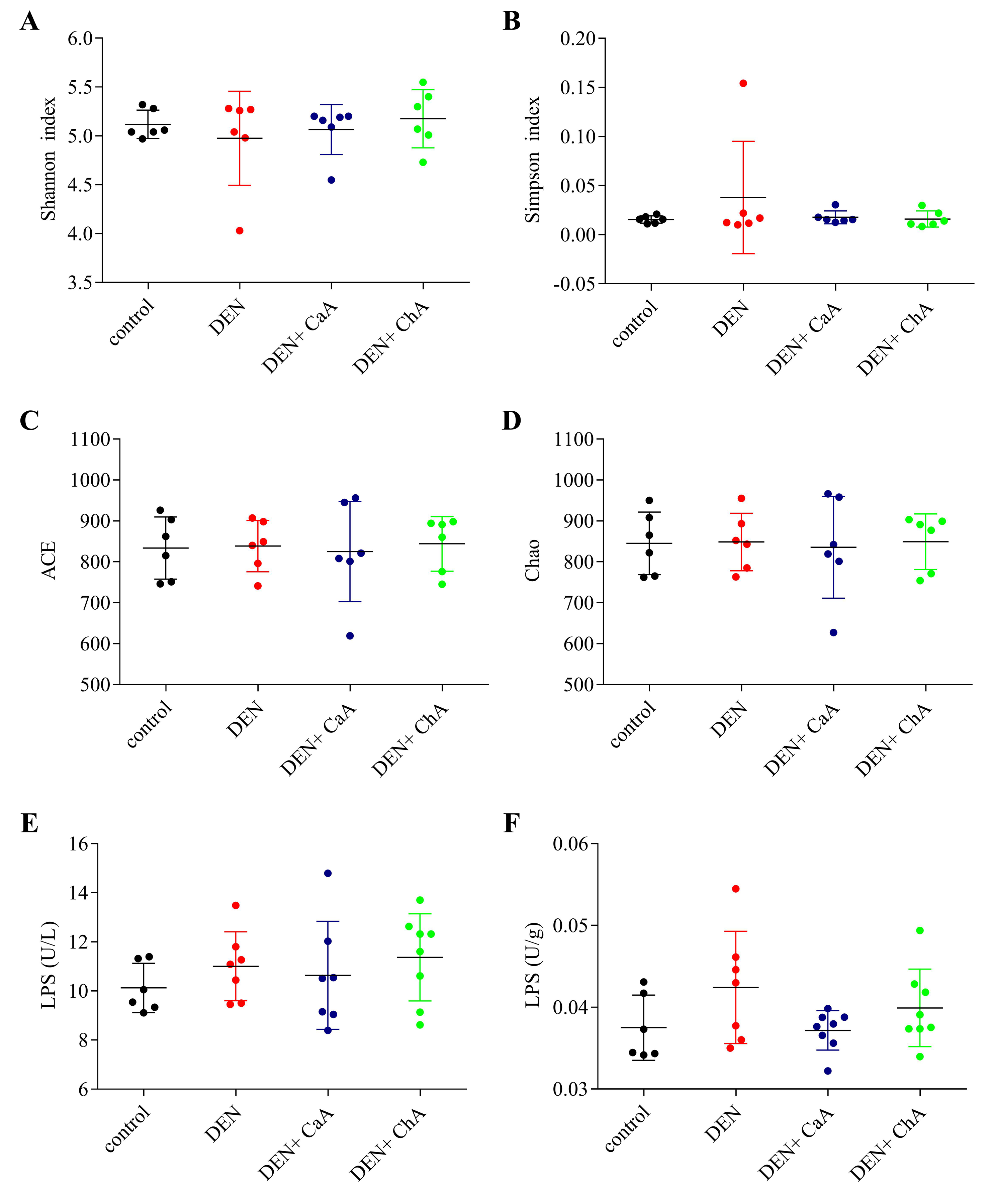
**

**Figure S1. The effects of CaA and ChA on fecal microbiota and LPS in HCC induced by DEN.** (A) Shannon index (B) Simpson index (AST), (C) ACE index, (D) Chao index, (E) serum LPS and (F) liver LPS.

**Table S1. The different metabolites among the control, DEN, CaA and ChA groups.**
